# Improving the Performance of Feather Keratin/Polyvinyl Alcohol/Tris(hydroxymethyl)Aminomethane Nanocomposite Films by Incorporating Graphene Oxide or Graphene

**DOI:** 10.3390/nano10020327

**Published:** 2020-02-14

**Authors:** Shufang Wu, Xunjun Chen, Tiehu Li, Yingde Cui, Minghao Yi, Jianfang Ge, Guoqiang Yin, Xinming Li, Ming He

**Affiliations:** 1 Green Chemical Engineering Institute, Zhongkai University of Agriculture and Engineering, Guangzhou 510225, China; SFWu2018@163.com (S.W.); MHYi0848@163.com (M.Y.); ge650704@163.com (J.G.); yingq007@163.com (G.Y.); lixinming@sina.com (X.L.); heming1026@163.com (M.H.); 2 Shaanxi Engineering Laboratory of Graphene New Carbon Materials and Applications, School of Materials Science and Engineering, Northwestern Polytechnical University, Xi’an 710072, China; litiehu@nwpu.edu.cn; 3 Guangzhou Vocational College of Science and Technology, Guangzhou 510550, China; 13602880087@139.com

**Keywords:** feather keratin, tris(hydroxymethyl)aminomethane, graphene oxide, graphene, solvent casting, nanocomposite

## Abstract

In this study, feather keratin/polyvinyl alcohol/tris(hydroxymethyl)aminomethane (FK/PVA/Tris) bionanocomposite films containing graphene oxide (GO) (0.5, 1, 2, and 3 wt%) or graphene (0.5, 1, 2, and 3 wt%) were prepared using a solvent casting method. The scanning electron microscopy results indicated that the dispersion of GO throughout the film matrix was better than that of graphene. The successful formation of new hydrogen bonds between the film matrix and GO was confirmed through the use of Fourier-transform infrared spectroscopy. The tensile strength, elastic modulus, and initial degradation temperature of the films increased, whereas the total soluble mass, water vapor permeability, oxygen permeability, and light transmittance decreased following GO or graphene incorporation. In summary, nanoblending is an effective method to promote the application of FK/PVA/Tris-based blend films in the packaging field.

## 1. Introduction

The awareness of sustainability is currently increasing, and the focus has shifted from conventional plastic materials to more environmentally friendly alternatives for different applications, such as food packaging. Therefore, the potential of keratin for use as a sustainable alternative to traditional petrochemical-derived polymers in packaging applications has received worldwide attention [1,2,3]. Keratin is one of the most abundant and underexploited sources of protein, is present in wool, hair, nails, hoofs, feathers, and horns (protein content 80–90%), and can be extracted from keratin-rich substances through physical- [4], chemical- [5,6], or microbial-based [7,8] methods. Keratin is a non-food-derived protein, so there is no issue of it becoming a source of competition for food with humans. The keratin structure has a large number of disulfide bonds; thus, keratin has good stability and low solubility. Moreover, keratin has good film-forming properties. However, pure keratin film without a plasticizer is very fragile. In our previous research, we found that blending feather keratin (FK), polyvinyl alcohol (PVA), and tris(hydroxymethyl)aminomethane (Tris) [9] prior to cross-linking modification of an FK-based film with CaCl_2_, transglutaminase, and genipin [10] can improve the mechanical properties, hydrophobicity, and thermal properties of keratin. However, the barrier properties of keratin-based blend films, particularly oxygen barrier properties, are not ideal. The barrier properties of the film materials directly affect their application in the packaging field. Therefore, other methods are needed to comprehensively improve the mechanical, thermal and barrier properties of keratin-based blend films.

Nanocomposite technology that uses low-filler-content nanofillers (e.g., carbon nanotubes, graphene, clay, and TiO_2_) has proven to be an effective way to produce new materials with customizable properties and high performance [11,12,13]. The mechanical, thermal, and barrier properties of the material can be significantly improved by adding a small amount of nanofillers to the system and ensuring that the nanomaterials are uniformly dispersed throughout the system. Additionally, polymer/graphene nanocomposites have attracted much attention because of their unique properties, which are not provided by traditional polymers [14]. Graphene, or monolithic graphite, is a two-dimensional carbon nanomaterial with a single-atom thickness [15,16]. Graphene has a high aspect ratio and specific surface area, high electrical conductivity, excellent mechanical properties, and good thermodynamic properties [17]. Ashori et al. [18] used the casting method to prepare graphene-reinforced chitosan/cassava starch blend films; they found that the addition of graphene improved the thermal stability, mechanical properties (e.g., the tensile strength increased by a maximum of 37.5%) and barrier properties (e.g., the water vapor transmission coefficient decreased by 12.6%) of the chitosan/cassava starch blend films.

Graphene oxide (GO) has good physical and chemical properties when implemented as a derivative and an important precursor of graphene; these properties include a light weight and high specific surface area [19]. GO contains a large number of oxygen-containing functional groups, such as hydroxyl, carboxyl, epoxy, and ester groups. The functional groups of the hydroxyl and epoxy groups exist on the surface of GO, whereas the functional groups of the carboxyl group exist on the edge. Therefore, GO has a large negative charge, and can be stably dispersed in water and partially polar organic solvents with a monolayer structure, which has better dispersion, hydrophilicity, and complexity [20,21]. Moreover, the combination of GO and polymer materials can yield composite materials with good electrical conductivity, mechanical and thermodynamic properties [22,23]. Ahmed et al. [22] employed a solution casting method to prepare a crab shell chitosan (CS)/GO nanocomposite film. A comparative analysis of the CS film loaded with 2 wt% GO and pure CS film revealed that incorporating GO yielded the following results: the tensile strength increased by 119.2%, the glass transition temperature increased by 26 °C, the water vapor transmission coefficient decreased by 56.2%, the oxygen transmission coefficient decreased by 65.2%, and the ultraviolet (UV) blocking performance was improved. Yadav et al. [23] employed a simple solution mixing-evaporation method to prepare a series of GO/carboxymethyl cellulose/alginate (GO/CMC/Alg) nanocomposite films. When the GO loading was 1 wt%, the tensile strength and elastic modulus of the composite film respectively increased by 40% and 1128% relative to those of the pure CMC/Alg films. In addition, the GO/CMC/Alg nanocomposite films was found to possess a higher storage modulus (E’) than the CMC/Alg films.

Therefore, adding graphene nanosheets to the keratin-based composite film can effectively improve the thermal, mechanical, and barrier properties of the blend film to meet the needs of certain specialized packaging fields. It should also be noted that there are no reports on the study of graphene nanosheets to enhance keratin-based blend films. Thus, in this study, GO- and graphene-modified FK/PVA/Tris blend films were prepared by the solution casting method, and the effects of GO or graphene loading on the structure and properties of the blended nanocomposites were subsequently investigated.

## 2. Materials and Methods

### 2.1. Materials

PVA with a repeat unit number of 1700 (degree of alcoholysis = 99%) was purchased from Aladdin Ltd. (Shanghai, China). The Tris was supplied by Shanghai Ebene Chemical Reagent Co., Ltd. (Shanghai, China). The GO (content > 98%, number of layers: 1–2) and graphene (content > 98%, number of layers: <3) were purchased from Chengdu Organic Chemistry Co., Ltd., Chinese Academy of Sciences (Chengdu, China). The average diameter of the GO (as recorded by the company) was larger than 50 μm and was synthesized from high-purity natural flake graphite by a modified Hummers method [24]. The graphene is reduced GO, with an average diameter of 5 μm and a thickness of 1–3 nm (as recorded by the company). The chicken FK powder was prepared as described in our previous report [9]. Deionized water was employed as a solvent.

### 2.2. Preparation of 0.5% GO Dispersion

A GO dispersion was prepared by adding GO powder (0.5 g) and Tris (3 g) to deionized water (96.5 g) at room temperature under the conditions of continuous stirring for 1 h and ultrasonication for 2 h, followed by further continuous stirring for 24 h.

### 2.3. Preparation of the Nanocomposite Films

P-40-25 (FK-to-PVA weight ratio of 60:40, and a Tris content of 25 wt% relative to the total weight of FK and PVA), which was developed in our previous work [9], was selected as the control group. The nanocomposite films were prepared as follows: the extracted FK powder (1.2 g) and 6% PVA solution (13.33 g) were weighed in a beaker (the total solid mass of FK + PVA was set to equal 2 g), and a certain amount of 6% Tris solution and deionized water were added under the condition of 30 min of continuous stirring at 40 °C. Then, 2, 4, 8, or 12 g of 0.5% GO dispersion, or 0.01, 0.02, 0.04, or 0.06 g of graphene powder was added under the conditions of 3 h of continuous stirring at 40 °C, and in consideration of the total weight of the 0.5%, 1%, 2%, or 3% FK and PVA; the specific amount of each added substance is shown in Table 1; note that each substance was added such that the total mass of the final film-forming solution was 40 g. Ultrasonication was applied for 2 h, and continuous magnetic stirring was performed in a 40 °C fixed-temperature water bath for 1 h; then, the cooled mixed solution was poured into polypropylene dishes (15 cm × 18 cm) The nano-modified FK/PVA/Tris composite film was obtained by drying the specimens in a humidity chamber that was maintained at 35 °C and 50% relative humidity for 12 h. The nanocomposite films were respectively named x-GO and x-Graphene, where x refers to the weight percentage of nanoparticles relative to the total weight of the FK and PVA in the film. The resulting blended films were conditioned at 25 °C and 50% relative humidity for 48 h prior to testing.

### 2.4. Characterization

The surface and cross-sectional morphologies of the nanocomposite films (following liquid nitrogen freezing treatment) were imaged using scanning electron microscopy (SEM, EVO 18; Carl Zeiss, Jena, Germany). The structures of the GO and graphene were also analyzed by using an SEM. Prior to analysis, the specimens were coated with a thin film of gold for 45 s in order to make the sample conductive; they were then analyzed at an accelerating voltage of 10–12 kV. 

An infrared spectrometer (Spectrum 100, Perkin-Elmer, Fremont, CA, USA) was operated in the attenuated total reflectance mode to obtain the Fourier-transform infrared (FTIR) spectroscopy results for the GO, graphene, and nanocomposite films. The FTIR spectra were recorded in the 650–4000 cm^−1^ range with a 4-cm^−1^ resolution. 

Thermogravimetric analysis (TGA) was performed by operating a thermogravimetric analyzer (TGA2, Mettler Toledo, Switzerland) with a 10 °C·min^−1^ ramp between 40 °C and 700 °C under the protection of a nitrogen flow; the mass of each tested specimen was 3–5 mg. 

The mechanical strength of each specimen was comprehensively evaluated in accordance with the ASTM D 882 standard by using a microcomputer-controlled electronic universal testing machine (CMT6503, Shenzhen MTS Test Machine Company, Ltd., China) with a speed of 10 mm/min and fixture distance of 40 mm. The composite films were cut into 10-mm-wide, 75-mm-long strips using a cutter, and each strip thickness was measured by using a digital external micrometer (precision: 0.001 mm). The reported data on the elastic modulus, tensile strength, and elongation at break correspond to the respective averages of three strips from the same sample.

The total soluble mass (TSM) of the films in 25 °C water was obtained by employing the method described by Yao et al. [25], with slight modifications. Initially, the square specimens (40 mm × 40 mm) were preconditioned by drying them in a vacuum oven at 70 °C for 24 h; they were then cooled to room temperature, removed from the vacuum oven, and immediately weighed (*W*1). The preconditioned specimens were then immersed in a beaker containing deionized water (100 mL) that was sealed with plastic wrap and placed in a humidity chamber maintained at 25 °C for 24 h. The resulting wet specimens were dried once again in a vacuum oven at 70 °C for 24 h before being cooled to room temperature, removed, and immediately weighed again (*W*2). The reported data on the TSM correspond to the averages of three samples of the same film. The TSM values for the films were calculated by solving the following equation:

TSM (%) = (*W*1 − *W*2)/*W*1 × 100% (1)

The water vapor permeability (WVP) values for the films were measured by using a water-vapor transmittance tester (W3/030, Labthink, Ltd., Jinan, China). To begin, each tested film sample was cut into a circular shape with an area of 7.065 cm^2^ and set in the instrument. Then, the testing conditions were set as follows: warm-up time: 4 h; weighing interval: 2 h; testing temperature: 38 °C; and relative humidity across the film: 90–0%. The measurements were conducted in triplicate, and the average values were calculated.

The oxygen permeability (OP) values for the films were measured according to the recommendation of the GB/T 1038-2000 standard by using an oxygen permeability tester (VAC-VBS, Labthink, Ltd., Jinan, China). To begin, the tested film sample was cut into a circular shape with an area of 23.746 cm^2^ and set in the instrument. The testing gas pressure and upper and lower degassing time were respectively set as 1.01 × 10^5^ Pa and 4 h. The reported OP data correspond to the averages of three specimens for each type of film.

The transmittance of the films was measured by using a spectrophotometer (UV-1800, Shimadzu Corporation, Chengdu, China) and following the method described by He et al. [26]. Each composite film was cut into 10-mm-wide, 40-mm-long strips, and directly attached to the quartz cuvette. An empty quartz cuvette was employed as the blank control, and the measurement range was 200 to 800 nm. The transparency value (T) of the films was calculated by solving the following equation.

T = −(log T_600_)/*x* (2)

where T_600_ is the transmittance at 600 nm, and *x* is the film thickness (mm). The measurements were conducted in triplicate, and the average values were calculated.

## 3. Results and Discussion

### 3.1. Morphology of the Nanocomposite Films

**Figure 1:**
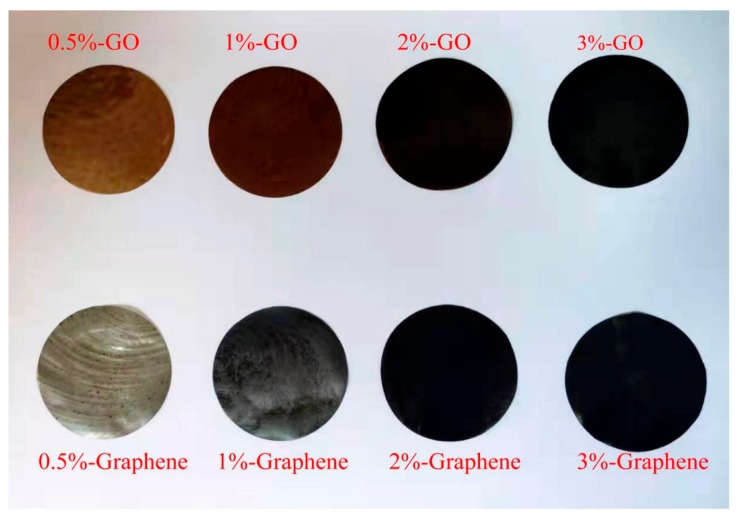
Photos of nano-blend films.

As shown in Figure 1, the surface of each type of nano-blend films was smooth, with no visible holes. The color of the GO-modified keratin-based blend films changed from brown to black as the GO content increased. Alternatively, increasing the amount of graphene caused the films to gradually darken. Additionally, the light transmittance of these two nano-modified blend films significantly decreased.

**Figure 2:**
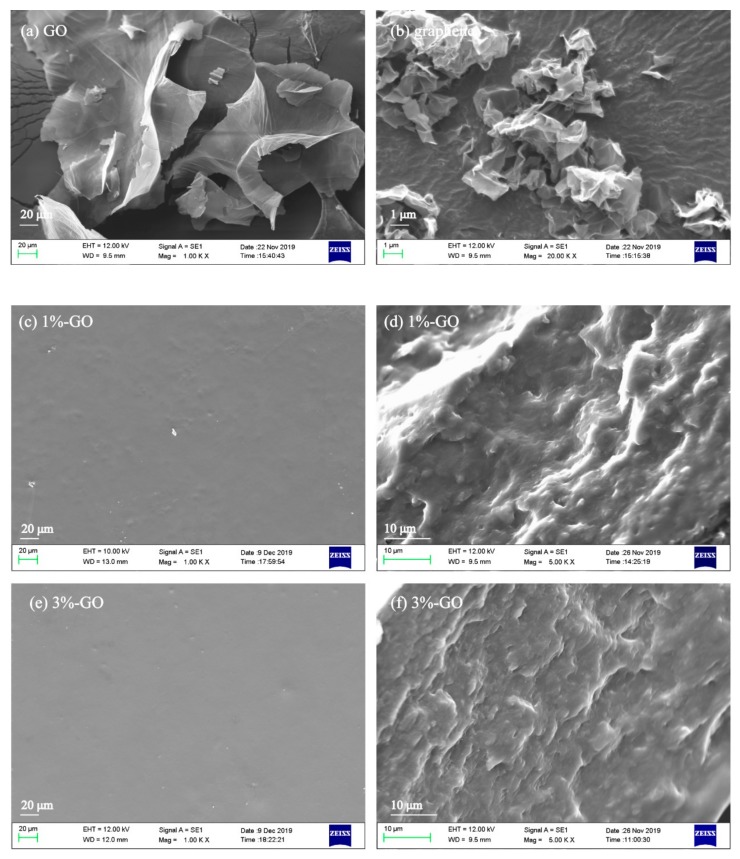

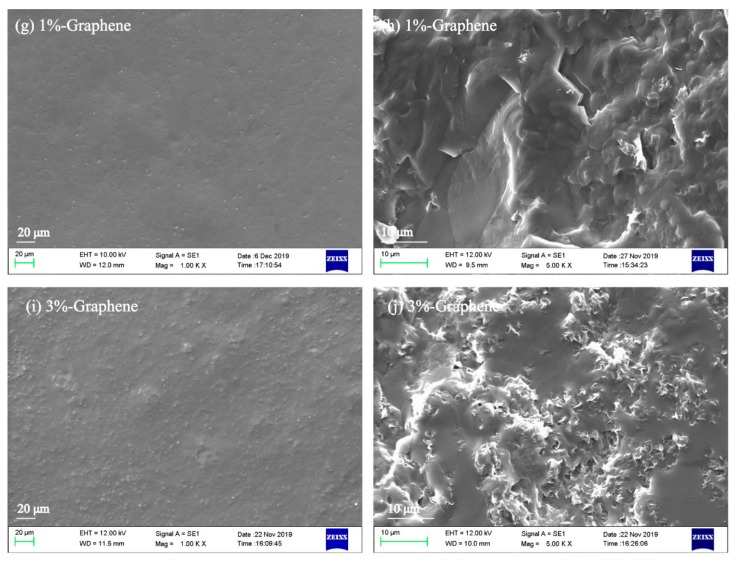
Scanning electron microscopy (SEM) images of (**a**) graphene oxide (GO) (1000×) and (**b**) graphene (20000×), and representative SEM images of the blend films: (**c**) 1%-GO surface morphology (1000×), (**d**) 1%-GO fracture morphology (5000×), (**e**) 3%-GO surface morphology (1000×), (**f**) 3%-GO fracture morphology (5000×), (**g**) 1%-Graphene surface morphology (1000×), (**h**) 1%-Graphene fracture morphology (5000×), (**i**) 3%-Graphene surface morphology (1000×), and (**j**) 3%-Graphene fracture morphology (5000×).

SEM images of the GO and graphene (purchased without any treatment) are provided in Figure 2a,b, respectively. As shown in the figure, the GO generally appeared as a single layer, and demonstrated good light transmission performance. However, the graphene appeared to as a multi-layer structure, as many folds can be observed in the layer.

To better understand the dispersion uniformity of the GO and graphene nanosheets in the blend films and the effects of their addition on the microstructure of the blend films, the surface and cross section of the 1%-GO, 3%-GO, 1%-Graphene, and 3%-Graphene nanocomposite films were analyzed by SEM. It can be seen in Figure 2c,e that the surface of the GO-modified FK/PVA/Tris blend films was smoother than that of the P-40-25 film (an SEM image of P-40-25 can be seen in our previous report [10]). Additionally, there were no visible granular protrusions, indicating that the GO was well dispersed throughout the film matrix (i.e., no aggregation), and that the filler was strongly adhered to the matrix. This may be attributable to the strong hydrogen bonding and electrostatic interaction between the GO and film matrix, which consequently inhibit phase separation. It can be seen in Figure 2g,i that the surface of the graphene-modified blend films was rough and had granular protrusions, and that these granular protrusions were generally uniformly distributed across the surface of the blend film, with no visible large-particle aggregation. Additionally, an increase in the graphene nanosheet content was observed to coincide with more granular protrusions in the film matrix. These granular protrusions are referred to as protruding graphene sheets, which are enveloped and thickly coated by a film matrix. Their occurrence indicates that, although the graphene nanosheets were firmly adhered to the film matrix, their dispersion uniformity in the film matrix was not as good as that of GO. Analysis of the cross section allowed for a more accurate assessment of the uniformity of the nanoparticle dispersion in the blend film. It can be ascertained from the cross-sectional structures shown in Figure 2d,f,h,j that the addition of nanoparticles tends to increase the roughness of the cross section; additionally, no large-particle aggregates were visible in the cross-sectional images, indicating that the GO and graphene were uniformly dispersed throughout the blend film.

### 3.2. FTIR Analysis

**Figure 3:**
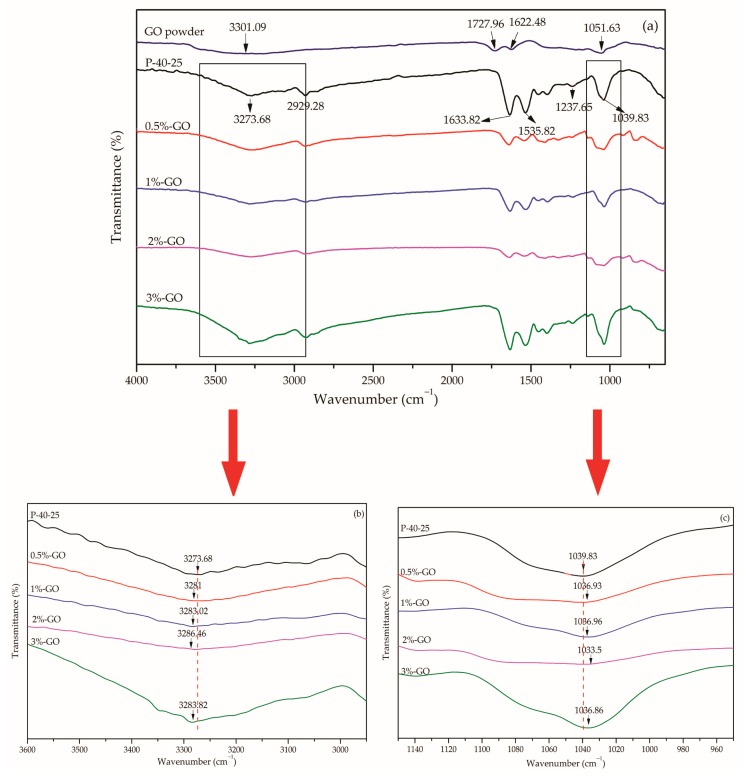

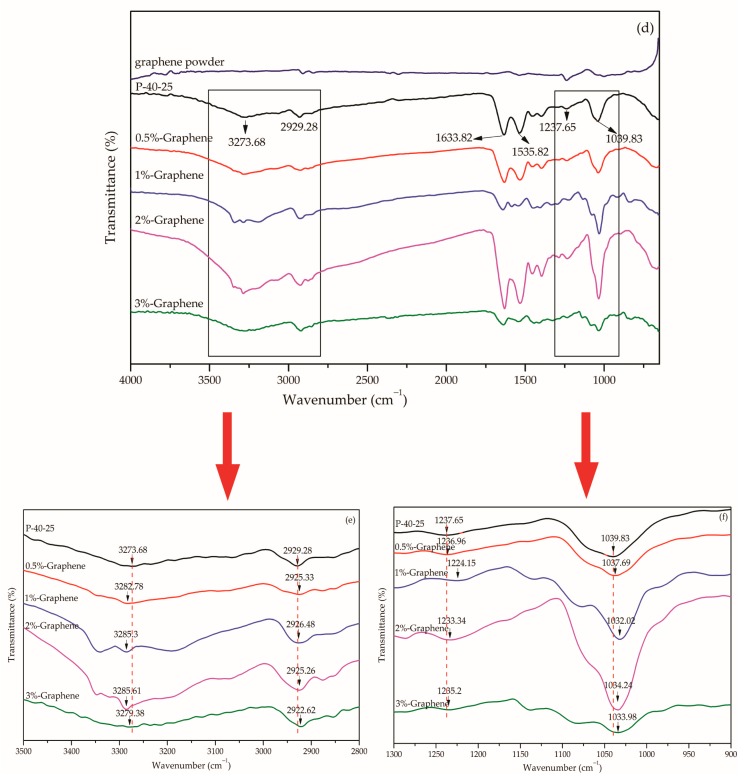
Fourier-transform infrared (FTIR) spectra for (**a**) GO powder and P-40-25 incorporated with GO in the region of 4000–650 cm^−1^, (**b**) P-40-25 incorporated with GO in the region of 3600–2900 cm^−1^, (**c**) P-40-25 incorporated with GO in the region of 1150–950 cm^−1^, (**d**) graphene powder and P-40-25 incorporated with graphene in the region of 4000–650 cm^−1^, (**e**) P-40-25 incorporated with graphene in the region of 3500–2800 cm^−1^, and (**f**) P-40-25 incorporated with graphene in the region of 1300–900 cm^−1^.

Figure 3a,d shows the FTIR spectrum of pure GO and graphene in the wavelength range of 4000–650 cm^−1^, respectively. Regarding the GO results, the 3301.09, 1727.96, and 1051.63 cm^−1^ peaks correspond to -OH stretching vibration, C=O stretching vibration, and C–O stretching vibration absorption, respectively. The absorption peak at 1622.48 cm^−1^ could be associated with the deformation of the OH band of the water absorbed by GO, or stretching of the C=C groups [27,28]. Alternatively, all the peaks in the graphene spectrum were found to be insignificant.

To clearly elucidate the interaction between the FK/PVA/Tris blend film and nanoparticles, the FTIR spectra of different nano-modified FK/PVA/Tris blend films were analyzed (Figure 3). It can be seen that the infrared spectrum of each nano-modified blend film significantly differs from that of the P-40-25 film, and that the peak positions are shifted. When 3%-GO was added to the blend film, the 3273.68- and 1039.83-cm^−1^ FTIR peaks for the P-40-25 control film, which respectively correspond to O–H and N–H association, and C–O stretching vibration [10], shifted to 3283.82 and 1036.86 cm^−1^, respectively (Figure 3b,c). These peak position shifts indicate that GO interacts with the film matrix through intermolecular hydrogen bonding; thus, it should have good miscibility with the film matrix [27,29]. When 3%-Graphene was added to the blend film, the 3273.68-, 2929.28-, 1237.65-, and 1039.83-cm^−1^ FTIR peaks for the P-40-25 control film [10], which respectively correspond to O–H and N–H association, –CH stretching vibration, amide III bands, and C–O stretching vibration, shifted to 3279.38, 2922.62, 1235.2, and 1033.98 cm^−1^, respectively (Figure 3e,f). These peak shifts indicate that the addition of graphene hinders the hydrogen bonding of the molecular chain of the film matrix at close vicinity of the graphene surface [30,31], and that there is strong interaction between the graphene and film matrix molecules that is mediated by van der Waals forces.

### 3.3. TGA

**Figure 4:**
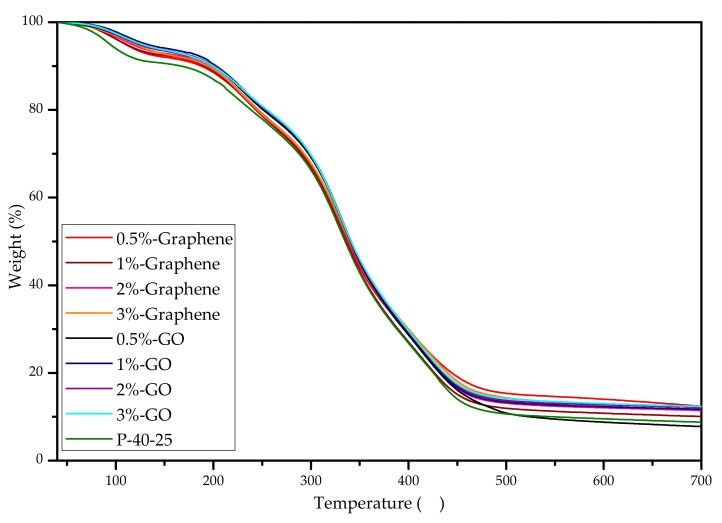
Thermogravimetric curves for P-40-25 incorporated with GO and graphene.

TGA was performed to characterize the thermal properties of the GO and graphene nanocomposite films (Figure 4). All blend films were found to have two mass-loss stages. The first mass loss stage (△1) occurred between 40 and 200 °C, and is primarily attributable to the loss of free water and bound water molecules [32]. The second mass loss stage (△2) occurred between 200 and 500 °C, and is mainly related to the degradation behavior of the mixture, i.e., this phase is accompanied by decomposition during the melting process [33]. This phenomenon is similar to the thermal degradation processes of sodium montmorillonite (MMT) and TiO_2_ nano-blend films [13]. The temperatures associated with the different degradation stages and the final residual amounts are listed in Table 2; the thermal data for P-40-25 were taken from our previous study [13]. In the first stage, when the mass loss reached 10%, T_d1_ for the nanocomposite film was 20 to 36 °C higher than that for the P-40-25 film. This can be attributed to the barrier effect of the nanomaterial, as the nanosheets can form a protective physical barrier on the surface of the material, thereby preventing water vapor from escaping from the nano-blend films; these conditions result in the nano-blend film experiencing the same amount of water vapor evaporation loss at higher temperatures [34,35]. During the second stage, T_onset_ for the nanocomposite films was significantly better than that for the P-40-25 film. Specifically, T_onset_ increased by approximately 15 °C, whereas T_max_ did not significantly change. It should also be noted that the final residual amount was larger. These results demonstrate that the addition of nanoparticles can improve the thermal stability of FK/PVA/Tris blend films through the nanoparticle barrier effect. Furthermore, the strong interfacial adhesion between the nanoparticles and film matrix was also found to hinder the migration of the molecular chain of the film matrix near the surface of the nanoparticles, thereby providing the films with excellent thermal stability [36].

### 3.4. Mechanical Properties

Table 3 lists the elastic modulus, tensile strength, and elongation-at-break values for the FK/PVA/Tris blend films prepared with different contents of GO and graphene. The tensile properties for the P-40-25 film have been reported by Chen et al. [9]. The elastic modulus and tensile strength of the blend film increased with increasing GO content; specifically, the elastic modulus increased by 62.6%, from 416.78 MPa (P-40-25) to 677.69 MPa (3%-GO). The tensile strength increased by 53.86%, from 9.58 MPa (P-40-25) to 14.74 MPa (3%-GO), and the elongation at break decreased by 76.92%. A similar enhancement in the tensile properties of biopolymers incorporated with GO has been reported by various researchers [22,23]. These results indicate that GO can increase the strength and stiffness of FK/PVA/Tris blend films at the expense of flexibility. The improvement in the mechanical properties is attributable to the high stiffness and aspect ratio of the nanosheets, and the strong hydrogen bonding interaction between the matrix and nanosheets, which consequently increases the elastic modulus and tensile strength [37]. Strong interaction between the film matrix and hydrophilic GO at the interface have been reported to more effectively promote load transfer from the matrix to the nanoparticles [38,39]. Moreover, a large number of intermolecular hydrogen bonds were observed to have formed between the GO and FK/PVA/Tris film matrix, resulting in strong interaction between the components that has been reported to hinder molecular chain slippage and reduce the elongation at break of the FK/PVA/Tris blend film [23]. 

It can be ascertained from Table 3 that increasing the graphene nanosheet content coincided with increases in the elastic modulus and tensile strength of the blend film. Specifically, the elastic modulus increased by 50.79%, from 416.78 MPa (P-40-25) to 628.47 MPa (3%-Graphene), and the tensile strength increased by 15.87%, from 9.58 MPa (P-40-25) to 11.1 MPa (3%-Graphene). These increases occurred because the addition of rigid graphene nanosheets improved transfer of the film matrix load, consequently increasing the elastic modulus and tensile strength of the FK/PVA/Tris blend film. Conversely, the elongation at break of the blend film initially increased, and then decreased; this may have occurred because of strong interfacial interaction between the graphene and film matrix, as confirmed by infrared spectroscopy and SEM, improving the load transfer from the film matrix to the graphene nanoparticles. In other words, the film matrix-nanoclay interface is not easily damaged during tensile deformation, thereby the elongation at break of the nano-blend film increased. As the graphene nanosheet content was continuously increased, the elongation at break of the nano-blend film decreased. This decrease may have been a consequence of the graphene content increase, as the phenomenon of confinement of graphene to the surrounding polymers is enhanced by intermolecular forces; this constrains the mobility and flexibility of the molecular chain of the membrane matrix.

When the nanosheet content was ≤1 wt%, the elastic modulus, tensile strength, and elongation-at-break values for the graphene-modified blend films were higher than those resulting from GO modification. Alternatively, when the nanosheet content exceeded 1 wt%, the elastic modulus and tensile strength values for the graphene-modified blend films were lower than those for the GO-modified blend films, but the elongation-at-break was higher than that for the GO-modified blend films. This phenomenon may be related to the mechanical strength of the nanoparticles, the compatibility of the nanoparticles with the membrane matrix, and their dispersibility in the film matrix [14]. When the nanoparticle load is small (i.e., ≤1 wt%), the addition of graphene nanosheets will significantly improve the mechanical properties of the FK/PVA/Tris blend film, because the mechanical strength of graphene is stronger than GO; however, when the nanoparticle load is large (i.e., >1 wt%), since the average diameter of graphene is smaller than GO, and its compatibility with the film matrix is not as good as GO, the dispersibility of graphene in the film is not as good as GO, the tensile properties of graphene-modified blend films are weaker than GO-modified blend films. 

### 3.5. TSM Results for the Blend Films

**Figure 5:**
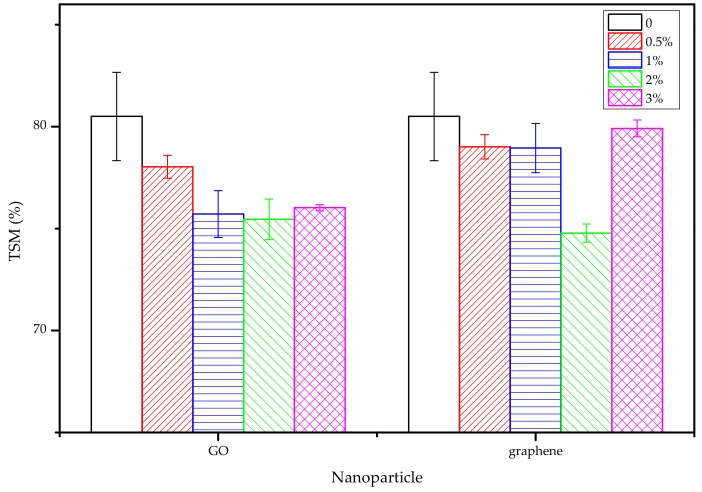
Total soluble mass (TSM) results for the blend films.

Figure 5 shows the TSM values for the P-40-25 film incorporated with different amounts of GO and graphene nanosheets. The results show that the addition of these two types of nanosheets can effectively reduce the TSM of the FK/PVA/Tris blend film, and that the TSM values for the blend film decreased with increasing nanoparticles content, i.e., the percentage decreased from 80.5% (P-40-25) [10] to 75.45% (2%-GO) and 74.78% (2%-Graphene), respectively. These results indicate that nano-modification can reduce the water sensitivity of the blend film and improve the stability of the FK/PVA/Tris blend film. These changes may be related to the strong hydrogen bonding between the GO nanosheets and film matrix, and the relatively lower hydrophilicity of the graphene nanosheets. When the GO content was increased to 3%, the water solubility of the film may increase due to the large number of oxygen-containing functional groups of GO. When the graphene content was increased to 3%, the increase in TSM may be due to the aggregation of graphene, which weakens the intermolecular forces between graphene and the film matrix.

### 3.6. Barrier Properties of the Blend Films

In the polymer packaging industry, especially in the food industry, the key factors determining the performance of the polymer films are the barrier properties, which are optimized for water vapor, oxygen, and UV rays [40]. Therefore, in this study, the barrier properties of nano-blend films were investigated, with focus on water vapor, oxygen, and UV light. 

#### 3.6.1. WVP

**Figure 6:**
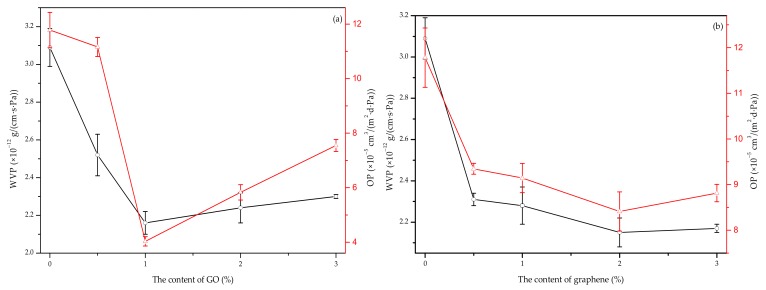
Water vapor permeability (WVP) and oxygen permeability (OP) values for (**a**) P-40-25 incorporated with GO and (**b**) P-40-25 incorporated with graphene.

Figure 6a shows the WVP results for the P-40-25 film loaded with varying levels of GO content. As the GO content was increased, the WVP values for the blend films decreased. A GO content of 1% yielded a minimum WVP value of 2.16 × 10^−12^ g·cm^−^^1^·s^−^^1^·Pa^−^^1^, which is 30.1% lower than that for the P-40-25 film [9]. The flake-shaped nanoparticles dispersed throughout the film matrix were determined to act as a physical barrier that prevents water molecules from penetrating the film. In addition to physical barriers, the interaction between the organic phase and the filler may also play the role of a barrier [41]. The synergistic adhesion at the film matrix-filler interface is primarily enabled by a wide range of hydrogen bonds, which limits spatial movement of the polymer chain, resulting in a very tight and dense network [42]. Upon increasing the GO content, a slight increase in the WVP value was observed, but this value remained lower than that for the control film. It was reported that the WVP process depended on the simultaneous actions of water diffusivity and solubility in a polymeric matrix [43]. Therefore, GO contains a large number of hydrophilic groups, which may lead to an increase in the solubility of water in the film, increasing the WVP values of 2%-GO and 3%-GO.

Figure 6b shows the WVP values for P-40-25 film loaded with different levels of graphene nanoparticle content. As the graphene content was increased, the WVP values for blend film initially decreased, and then slightly increased; the WVP values for all graphene nanocomposite films were lower than the corresponding values for the P-40-25 values [9]. A graphene content of 2% yielded a minimum WVP value of 2.15 × 10^−12^ g·cm^−^^1^·s^−^^1^·Pa^−^^1^, which is 30.42% lower than that for the P-40-25 film [9]. The incorporation of nanosheets has been reported to the enhance the barrier properties of polymer-based nanocomposites through the “curved path effect” [44]. When the graphene content was increased to 3%, the WVP value of the nano-blended film increased slightly. The possible reason was that the graphene partially aggregated and weakened the nano-barrier effect. A similar result was previously observed in the chitosan and tapioca starch blend films filled with graphene [18].

These results show that the addition of GO and graphene improved the water-vapor barrier performance of the blend films, and that the two types of nanosheets demonstrate better water-vapor barrier performance than films with MMT or TiO_2_ [13]. Moreover, the addition of graphene nanosheets most significantly improved the water-vapor barrier performance of the blend film. A possible reason for this is that, as compared to graphene, the surface of GO, MMT, and TiO_2_ contains more hydrophilic groups, which makes the three-nanoparticle-modified blend films more compatible with water vapor molecules; thus, their water-vapor barrier performance is not as good as that of graphene-modified FK/PVA/Tris blend films [45].

#### 3.6.2. OP

The OP values for P-40-25 film with varying levels of GO content are also shown in Figure 6a. The OP values for the blend films initially decreased, and then slightly increased, as the GO content was increased; this trend was also observed in the WVP results. When the GO content was set to equal 1%, the OP of the nanocomposite film reached a minimum value of 4.032 × 10^−5^ cm^3^·m^−2^·d^−1^·Pa^−1^, which is 65.77% lower than that for the P-40-25 film [9]. The gas barrier property improvement is attributable to the excellent impermeability of GO, and the strong interfacial adhesion between the GO and film matrix [44].

The OP values for P-40-25 incorporated with varying levels of graphene content are also shown in Figure 6b. The OP values for the blend films initially decreased, and then slightly increased, as the graphene content was increased; this trend was also observed in the WVP results. When the graphene content was set to equal 2%, the OP of the nanocomposite film reached a minimum value of 8.412 × 10^−5^ cm^3^·m^−2^·d^−1^·Pa^−1^, which is 28.59% lower than that for the P-40-25 film [9].

Therefore, the addition of GO or graphene nanosheets can improve the oxygen barrier performance of keratin-based blend films. This may be because of the strong interaction between the nanosheets and film matrix. Moreover, small-sized nanoparticles tend to occupy the voids of the porous film matrix. This can reduce the free volume of the film matrix, interfere with the transmission of oxygen through the film matrix, and extend the path of oxygen diffusion through the film matrix, thereby reducing the amount of oxygen transmission [46]. The oxygen barrier performance of FK/PVA/Tris blend films with MMT, TiO_2_, GO, or graphene addition was found to be ordered as follows: MMT nano-blend film > GO nano-blend film > graphene nano-blend film > TiO_2_ nano-blend film [13]. This finding indicates that a FK/PVA/Tris blend film modified by sheet nanoparticles has better oxygen barrier properties than the spherical TiO_2_-modified blend film. Furthermore, the dispersibility of MMT and GO in the film matrix is better than that of graphene. This may be the reason why the oxygen barrier performance of MMT- and GO nano-modified blend films is better than that for graphene nanocomposite films.

#### 3.6.3. Light Transmission and Transparency

Table 4 lists the transmittance values for the GO and graphene nano-modified FK/PVA/Tris blend films at selected wavelengths ranging between 200 and 800 nm; note that the transmittance data for the P-40-25 film were acquired from our previous study [13]. The results revealed that an increase in nanoparticle content corresponded to a decrease in the transmittance of the blend film at all wavelengths; moreover, the amount of reduction varied according to the nanoparticle type and content. For GO-modified films, the transmittance sharply decreased with decreasing wavelength, whereas for graphene-modified blend films, decreasing the wavelength yielded a more subtle change in transmittance. Furthermore, the light transmittance of the FK/PVA/Tris blend film decreased with increasing nanoparticle content. The light transmittance may have decreased because of the light-scattering or light-absorption effect of the nanoparticles that were distributed throughout the film matrix [47]. It is worth noting that, when the nanoparticle content was 1 wt%, the transmittance in the UV region (350 nm) decreased from 18.4% of that of the P-40-25 film, to 0.27% and 4.01% of that of the 1%-GO and 1%-Graphene films, respectively. These results indicate that the excellent UV-shielding characteristics associated with such a low nanoparticle content allow nanocomposite films to be used as packaging materials to protect perishable items that are subject to high-energy light degradation [48]. Therefore, the addition of GO or graphene can improve the UV barrier properties of FK/PVA/Tris blend films.

**Figure 7:**
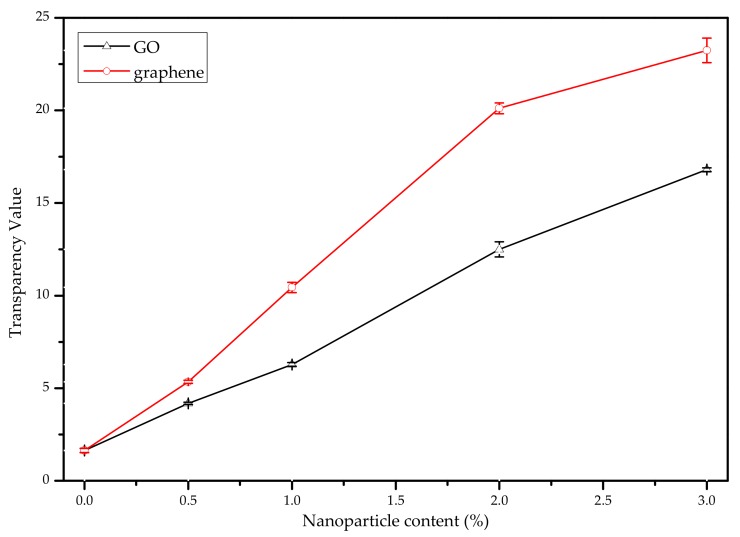
Transparency value (T) of the P-40-25 film modified with varying levels of GO and graphene content.

It can be seen in Figure 7 that the transparency values for the nano-modified blend films were higher than those for the P-40-25 film, and that the transparency value increased with increasing nanoparticle content. A similar trend in transparency was observed by Mahmoudi et al. [49]. At the same nanoparticle content, the GO nano-blend film was found to have a transparency value that is lower than that for the graphene nano-blend film and TiO_2_ nano-blend film, but higher than that for the MMT nano-blend film [13]. Specifically, a higher transparency value was found to correspond to a lower film transparency. The highest-to-lowest transparency of FK/PVA/Tris blend films with MMT, TiO_2_, GO, or graphene was as follows: P-40-25 > MMT nano-blend film > GO nano-blend film > TiO_2_ nano-blend film > graphene nano-blend film. Film transparency is typically affected by additives, processing conditions, thickness, and the compatibility between the polymers and nanoparticles [50,51,52,53,54]. The limited compatibility between the FK/PVA/Tris film matrix and nanoparticles, especially at higher nanoparticle contents, may induce large agglomerated nanoparticle phases, making the film more internal light scattering and becoming turbid [55]. Therefore, the addition of nanoparticles affects the appearance and photoresistance of FK/PVA/Tris blend films, the performance of which is strongly dependent on the nanoparticle type and content.

## 4. Conclusions

In this study, we successfully prepared FK/PVA/Tris nanocomposite films with GO or graphene. SEM analysis revealed that the dispersion of GO in the film matrix is better than that of graphene. The characteristic peak shifts that were observed in the FTIR spectra and those that occurred as a result of incorporating GO into the P-40-25 film have been associated with the formation of new hydrogen bonds in the nanocomposite matrix. It was observed that incorporating GO or graphene into FK/PVA/Tris blend films yielded with better thermal, mechanical, water resistance, and barrier properties than the unfilled material. Specifically, the P-40-25 film loaded with 3 wt% of GO or graphene demonstrated the most significant improvement in tensile properties, as the tensile strength increased by 53.86% (3%-GO) and 15.87% (3%-Graphene), and the elastic modulus increased by 62.6% (3%-GO) and 50.79% (3%-Graphene). Additionally, for the P-40-25 film loaded with 1 wt% of GO, the most significant improvements in barrier properties were as follows: the WVP and OP, respectively, decreased by 30.1% and 65.77% relative to the corresponding values for the P-40-25 film. Furthermore, for the P-40-25 film loaded with 2 wt% graphene, the most significant improvements in barrier properties were as follows: the WVP and OP, respectively, decreased by 30.42% and 28.59% relative to the corresponding values for the P-40-25 film. It is worth noting that the addition of GO or graphene was found to improve the ultraviolet resistance of the films. If GO is functionalized (e.g., with amines or other groups), the dispersion as well as the mechanical properties and conductivity of the materials will be significantly increased. Thus, this study was able to yield a method to improve the properties of keratin-based films for packaging applications.

## Figures and Tables

**Table 1:** Amount of each component in the blend films.

Sample	FK Powder (g)	6% PVA (g)	6% Tris (g)	0.5% GO (g)	Graphene (g)	H2O (g)
P-40-25	1.2	13.33	8.33	0	0	17.14
0.5%-GO	1.2	13.33	7.33	2	0	16.14
1%-GO	1.2	13.33	6.33	4	0	15.14
2%-GO	1.2	13.33	4.33	8	0	13.14
3%-GO	1.2	13.33	2.33	12	0	11.14
0.5%-Graphene	1.2	13.33	8.33	0	0.01	17.13
1%-Graphene	1.2	13.33	8.33	0	0.02	17.12
2%-Graphene	1.2	13.33	8.33	0	0.04	17.1
3%-Graphene	1.2	13.33	8.33	0	0.06	17.08

**Table 2:** Thermogravimetric analysis (TGA) results for P-40-25 incorporated with graphene oxide (GO) and graphene.

Sample	△1	△2		Residue (%)
	T_d1_ (°C)	T_onset_ (°C)	T_max_ (°C)	
P-40-25	167.5	214.83	329.33	8.75
0.5%-GO	200.33	229	331.33	7.77
1%-GO	203	230	331.17	11.74
2%-GO	199.33	231.5	329.33	12.18
3%-GO	200	231.83	328.83	12.37
0.5%-Graphene	187.83	234.17	328.83	12.4
1%-Graphene	191.67	230.67	329.5	10.08
2%-Graphene	195.17	230.67	330.67	11.45
3%-Graphene	194.17	229.33	329.17	12.11

△1 is the first mass loss stage. △2 is the second mass loss stage. T_d1_ is the temperature at which the mass loss is 10%, T_onset_ is the initial second-stage degradation temperature, and T_max_ is the fastest degradation temperature in the second stage, which corresponds to the peak value of the first derivative of the thermogravimetric curve.

**Table 3:** Tensile properties of the blend films.

Sample	Elastic Modulus (MPa)	Elongation at Break (%)	Tensile Strength (MPa)	Thickness (mm)
P-40-25	416.78 ± 17.34	10.83 ± 1.01	9.58 ± 0.37	0.087 ± 0.004
0.5%-GO	417.73 ± 16.57	10.58 ± 1.06	9.79 ± 0.29	0.0956 ± 0.008
1%-GO	425.72 ± 7.97	6.92 ± 0.34	10.00 ± 0.44	0.0905 ± 0.006
2%-GO	570.81 ± 12.08	4.5 ± 0.4	12.96 ± 0.22	0.0967 ± 0.007
3%-GO	677.69 ± 8.73	2.5 ± 0.22	14.74 ± 0.32	0.0956 ± 0.003
0.5%-Graphene	419.76 ± 15.94	11.62 ± 0.85	10.07 ± 0.31	0.0892 ± 0.008
1%-Graphene	426.76 ± 11.69	9.65 ± 0.88	10.55 ± 0.12	0.0977 ± 0.016
2%-Graphene	485.87 ± 20.3	7.78 ± 0.57	10.93 ± 0.32	0.0939 ± 0.007
3%-Graphene	628.47 ± 12.25	4.41 ± 0.16	11.1 ± 0.31	0.0861 ± 0.004

**Table 4:** Transmittance of the P-40-25 film modified with varying levels of GO and graphene content.

Sample	%T								
	800 nm	700 nm	600 nm	500 nm	400 nm	350 nm	300 nm	280 nm	200 nm
P-40-25	79.65	77.57	74.21	66.73	41.49	18.40	0.67	0.10	0.00
0.5%-GO	61.43	54.04	44.20	30.73	11.76	4.60	0.45	0.00	0.00
1%-GO	45.00	34.38	22.39	10.35	1.60	0.27	0.00	0.00	0.00
2%-GO	23.29	13.51	5.69	1.24	0.04	0.00	0.00	0.00	0.00
3%-GO	16.69	8.42	2.89	0.46	0.02	0.00	0.00	0.00	0.00
0.5%-Graphene	36.66	34.80	32.15	27.47	15.12	7.75	0.59	0.00	0.00
1%-Graphene	15.19	14.46	13.43	11.62	6.93	4.01	0.35	0.00	0.00
2%-Graphene	1.97	1.84	1.66	1.37	0.73	0.38	0.00	0.00	0.00
3%-Graphene	1.08	1.04	0.95	0.79	0.41	0.25	0.00	0.00	0.00
